# The mediating role of meaning in life between experiential avoidance and death anxiety among cancer patients: a cross-sectional study

**DOI:** 10.1186/s12885-024-12433-0

**Published:** 2024-05-31

**Authors:** Yixia Yan, Yongyi Chen, Meijun Ou, Youwen Gong, Renting Yang, Xiangyu Liu, Wanting Xia, Furong Chen, Hongling Zheng, Xianghua Xu

**Affiliations:** 1 The Affiliated Cancer Hospital of Xiangya School of Medicine, Central South University/ Hunan Cancer Hospital, Changsha, Hunan China; 2 School of Nursing, University of South China, Hengyang, Hunan China; 3 Xiangya Nursing School, Central South University, Changsha, Hunan China

**Keywords:** Death anxiety, Meaning in life, Experiential avoidance, Cancer patients, Mediation effect

## Abstract

**Background:**

Death anxiety is thought to cause a range of mental disorders among cancer patients, which may affect their mental health and even quality of life. This study sought to investigate experiential avoidance, meaning in life, and death anxiety among Chinese cancer patients and then explore the relationship between these 3 variables.

**Methods:**

A total of 300 cancer patients recruited from a tertiary cancer hospital participated in this study from October to December 2021. A cross-sectional survey was conducted using a demographic and clinical characteristics questionnaire, the Acceptance and Action Questionnaire II, the Meaning in Life Questionnaire, and Templer’s Death Anxiety Scale. Correlation analysis, hierarchical regression analysis, and mediating effect analysis were used to analyze the relationship among experiential avoidance, meaning in life (including 2 dimensions: presence of meaning and search for meaning), and death anxiety.

**Results:**

A total of 315 questionnaires were distributed, and 300 valid questionnaires were returned, resulting in a valid response rate of 95.2%. Experiential avoidance (*r* = 0.552, *p* < 0.01) was moderately positively correlated with death anxiety. Presence of meaning (*r* = − 0.400, *p* < 0.01) was moderately negatively correlated with death anxiety, while search for meaning (*r* = − 0.151, *p* < 0.01) was weakly negatively correlated with death anxiety. Regression analysis showed that experiential avoidance (β = 0.464) and presence of meaning (β = −0.228) were predictors of death anxiety. Mediating effect analysis revealed that presence of meaning either completely or partially mediated the effect of experiential avoidance and death anxiety, and the indirect effect accounted for 14.52% of the total effect.

**Conclusion:**

Overall, experiential avoidance predicts death anxiety in cancer patients, and meaning in life can mediate this effect. The results of this study provide a new path for studying the mechanism of death anxiety and suggest a more positive and promising strategy for its management.

## Background

Cancer has become a major public health problem that threatens the health of the global population. The International Agency for Research on Cancer predicted that there would be about 19.3 million new cancer cases and 10 million deaths worldwide in 2020 [1]. The updated 2022 cancer report of the National Cancer Center of China shows that there are about 4,064,000 new cancer cases and 2,143,500 deaths in China each year [2]. Cancer is a life-threatening illness which also has a negative impact on people’s psychological wellbeing [3]. Most cancer patients experience uncertainties about the effectiveness of anti-cancer treatments, worries about the subsequent progression of their disease, and fears about tumor recurrence and metastasis, and they often regard cancer as a synonym for death. When cancer patients realize the inevitability of death, they are prone to experience anxiety, fear, and even a strong sense of meaninglessness and destruction [4]. In the field of psychology, this negative psychological state associated with death is known as death anxiety (DA) [5].

DA refers to a feeling of unsafety, anxiety, or fear related to the occurrence of death or near-death. As a conscious or unconscious negative psychological state, a series of defense mechanisms can be triggered when facing the threat of death. This kind of anxiety is mainly derived from worries about what happens to individuals after their death, concerns about the upcoming dying process, and fear of one’s non-existence [6]. DA is thought to be a transdiagnostic construct of many mental disorders [7]. Studies have shown that DA among cancer patients is at a high level, and 70–80% of cancer patients experience anxiety and distressing thoughts under the threat of death [8, 9]. The Asian population has higher rates of DA than those of populations in Europe and North America [10]. DA can affect patients’ confidence and cooperation during anti-cancer treatment, trigger psychological distress, and reduce their quality of life [11, 12]. Therefore, understanding the characteristics of DA and its correlations with other psychological variables can contribute to improving the mental health and quality of life of cancer patients.

Previous studies have revealed a correlation between DA and meaning in life (MIL). Steger et al. defined MIL as “the sense made of, and significance felt regarding, the nature of one’s being and existence” [13], then proposed a model of MIL composed of 2 dimensions: search for meaning and presence of meaning [14]. The search for meaning, a motivational dimension, emphasizes the process by which individuals actively identify meaning and purpose in their lives. In contrast, the presence of meaning, a cognitive dimension, concentrates on the outcomes of the degree to which a person is committed to a goal, objective, or mission in life; when individuals achieve these goals, they experience meaning in their lives. So far, most researchers have supported this 2-dimensional model [15]. Human beings have an innate motivation to find meaning and significance in their lives, and failure to achieve meaning in this regard leads to psychological distress [16]. It has been shown that MIL has a significant predictive effect on DA [17]. Individuals with low levels of MIL have a worse awareness and understanding of the meaning, mission, and value of life, and a higher degree of DA, which damages the physical and mental health of patients, influences the will to live, and affects the treatment effect, tumor process, and disease regression [18].

Another potential variable associated with DA is experiential avoidance (EA). EA can be defined as an attempt to avoid or ignore unpleasant thoughts, unpleasant feelings, painful memories, and uncomfortable physical sensations, leading to behaviors that run counter to one’s values and cause long-term harm [19]. Empirical evidence suggests that EA contributes to the psychopathology of cancer patients [20]. EA is a common response to a cancer diagnosis, prognosis, or treatment [21]. EA has been validated as one of the defenses of cancer patients and as a predictor of negative psychological symptoms, such as anxiety and distress [22]. Attempts to avoid unpleasant thoughts, feelings, and memories associated with death instead increase patients’ discomfort and DA, leading to their lack of engagement in worthwhile activities and life. In many studies, reducing EA can enhance patients’ psychological flexibility and thus promote their acceptance. Psychological flexibility is the ability to fully experience the present moment, emphasizing active acceptance rather than efforts to control or change one’s thoughts and feelings [19]. Having psychological flexibility is an important factor in preventing negative psychological problems, such as anxiety and depression, in cancer patients [23]. People with high psychological flexibility are able to free themselves from rigid thoughts and actions, enjoy the present moment, and accept things and circumstances that are beyond their control, thus enhancing their death acceptance reducing anxiety about death.

Existing theories provide hypotheses for the coping mechanisms of DA. At present, the defense-based terror management theory (TMT) remains the predominant theoretical explanation for our coping with DA, and in recent years the acceptance-based meaning management theory (MMT) has been gradually proposed to help explain the coping mechanisms of DA [24]. TMT assumes that humans have a self-protective instinct, and that their capacity for self-consciousness makes them terrified of their own mortality, then initiating proximal defenses (including EA). Subsequently, humans defend themselves against death anxiety through the distal defense mechanism of participating in cultural worldviews and deriving a sense of meaning and self-esteem from these worldviews (including MIL). MMT establishes an adaptive coping mechanism for DA from a positive value orientation. It argues that MIL is the essence of individual motivation, that death evokes a strong need for individuals to seek MIL, and that meaning comes from accepting death and committing to the pursuit of life expansion and self-transcendence [24].

It has been shown that that EA and MIL are important influencing factors in DA. Several studies have also confirmed the correlation between EA and MIL [25], and scholars have pointed out that MIL may buffer the impact of EA on DA [26]. However, the tripartite relationship and the degree of interaction between DA, EA, and MIL in cancer patients have not been clarified. Because of the distinctive characteristics of Chinese society, economy, and culture, the DA of cancer patients in China might exhibit unique features compared to other populations. Exploring the interactions among these three variables can provide a reference for strategies to reduce death anxiety in cancer patients and promote their physical and psychological wellbeing. The following hypotheses were proposed in this study: (1) Chinese cancer patients may experience a higher level of DA, and (2) EA is a predictor of DA, and (3) MIL mediates the association between EA and DA. This study will investigate EA, MIL, and DA among Chinese cancer patients, then confirm the mediating role of MIL in the relationship between EA and DA.

## Methods

### Study design

This study was a descriptive cross-sectional study performed using a self-administered questionnaire to collect data on factors related to DA, EA, and MIL among cancer patients.

### Participants and procedures

Participants were recruited by convenience sampling, and hospitalized cancer patients at a tertiary cancer hospital in Hunan Province between October and December 2021 were included. Patients considered eligible for inclusion were those who (1) were aged ≥ 18 years, (2) had a diagnosis of malignancy according to pathological results, (3) had normal cognitive function, (4) had an awareness of the diagnosis of the disease, and (5) voluntarily participated in this study and provided informed consent. Patients were excluded if they (1) had a mental illness or psychiatric disorder, (2) had communication or reading comprehension barriers, or (3) had activity intolerance or an inability to cooperate.

Of the 315 questionnaires distributed, 15 incomplete questionnaires were excluded because (1) rate of missing answers to survey questions was > 15% or (2) logic contradictions were found in the responses to the questionnaires. Finally, 300 valid questionnaires were included for analysis.

### Measures

#### Demographic and clinical information

Demographic information of interest included gender, age, nationality, education level, marital status, occupation, residence area, income, insurance, and religion. In addition, cancer position, cancer stage, and with or without pain were included as clinical information.

#### Experiential avoidance

Acceptance and Action Questionnaire II (AAQ-II) was a commonly used tool for evaluating EA, and it was originally developed by Bond et al. in 2011 [27]. The AAQ-II unidimensional scale includes 7 items scored using a 7-point Likert scale ranging from 1 point (never true) to 7 points (always true). During testing, the total score is summed over the 7 items, with higher scores representing greater EA. The Chinese version of the AAQ-II was translated by Cao et al. and was initially validated among Chinese college student in 2013 [28]. The translated version exhibited satisfactory psychometric properties, and it demonstrated acceptable internal consistency (Cronbach’s α = 0.88) and test-retest reliability (*r* = 0.80). The Cronbach’s α in this study was 0.94.

### Meaning in life

MIL was measured using the Meaning in Life Questionnaire (MLQ), which was developed by Steger [14]. The questionnaire contains 2 dimensions, as follows: (1) presence of meaning (MLQ-P), which assesses the extent to which meaning is experienced in a respondent’s life using statements such as “I understand my life’s meaning,” and (2) search for meaning (MLQ-S), which assesses a respondent’s desire to find and understand MIL using statements such as “I am searching for meaning in my life.” The original questionnaire has 10 items (5 items for each of the 2 subscales) scored using a 7-point Likert scale ranging from 1 point (absolutely untrue) to 7 points (absolutely true). Higher scores on MLQ suggest that respondents are more likely to perceive and find MIL. Chinese researchers have previously translated and revised the questionnaire, removing the original item “I am always searching for something that makes my life feel significant”, which cross-loaded in the exploratory factor analysis. Ultimately, 5 questions for MLQ-P and 4 questions for MLQ-S remain in the Chinese version of the questionnaire. This scale has been validated in university students and has satisfactory psychometric properties. The internal consistencies of MLQ-S and MLQ-P were 0.72 and 0.81, respectively [29], compared with 0.87 and 0.86 in this study.

#### Death anxiety

Templer’s Death Anxiety Scale (T-DAS) was developed by Templer in 1967 and is the most commonly used scale to evaluate the degree of DA in cancer patients [30]. The T-DAS scale consists of 15 items with dichotomous responses (true/false); of these, 9 items are forward-scored and 6 items are reverse-scored, respectively, so that higher scores consistently indicate greater DA. It has been translated into dozens of languages and used in multiple countries so far. The Chinese version of T-DAS was modified by Yang et al. and validated among medical students and hospice professionals [31]. Four factors were generated by principal component analysis in the Chinese version, which were as follows: (1) stress and pain, (2) emotion, (3) cognition, and (4) awareness of time passing. The translated measure demonstrated good reliability and validity, with an estimated internal consistency of Cronbach’s α = 0.71 and a test-retest reliability of *r* = 0.83. The Cronbach’s α in this study was 0.85.

### Data analysis

SPSS Statistics version 26.0 (IBM Corporation, Armonk, NY, USA) and SPSS Amos version 23.0 (IBM Corporation, Armonk, NY, USA) were used for data analysis. A *p*-value of < 0.05 was considered to be statistically significant. Descriptive statistics and Pearson’s correlation were used for statistical analysis to preliminarily identify relationships between MIL, EA, and DA. As recommended by Baron and Kenny’s causal steps approach [32] and Wen’s procedures for mediation effect analysis [33], hierarchical regression analysis was applied to test whether MIL mediated the relationship between EA and DA. The following procedure was carried out: first, we tested the total effect (the significance of c) of the independent variable on the dependent variable; second, we tested the effect (the significance of a) of the independent variable on the mediating variable; third, we tested the effect (the significance of b) of the mediating variable on the dependent variable; and fourth, if at least one of a and b was not significant, we used a mediating-effect analysis to test the mediating effect in IBM SPSS Amos version 23.0. The bootstrap test was performed to test the significance of indirect and direct effects in the mediating model. The model fit indices were as follows [34, 35]: relative chi-square (χ2/ df), root mean square error of approximation (RMSEA), goodness-of-fit index (GFI), normed fit index (NFI), incremental fit index (IFI), Tucker–Lewis index (TLI), and comparative fit index (CFI).

## Results

### Patient characteristics

A total of 315 questionnaires were distributed, and 300 valid questionnaires were returned, resulting in a valid response rate of 95.2%. Demographic and clinical details of the 300 Chinese cancer patients are presented in Table 1. The age of the participants ranged from 22 to 89 years, with a mean ± standard deviation age of 56.35 ± 11.02 years. Among the study participants, 49.7% were male and 50.3% were female, and most were Han (88.3%), married (88.3%), currently employed (72.0%), and non-religious (94.0%). More than half of the participants were from rural areas (56.3%) and had new rural cooperative medical insurance (56.0%). In addition, a significant number of participants had stage II (34.0%) or stage III (46.3%) cancer, and more than half of the participants reported experiencing pain (58.0%).

**Table 1:** Demographic and clinical characteristics (*n* = 300)

Variables	Groups	*n* (%)
Gender	Male	149 (49.7)
	Female	151 (50.3)
Age (years)	< 40	26 (8.7)
	40–60	172 (57.3)
	> 60	102 (34.0)
Nationality	Han	265 (88.3)
	National minority	35 (11.7)
Educational level	Primary school or below	89 (29.7)
	Middle school	123 (41.0)
	High school	45 (15.0)
	College or above	43 (14.3)
Marital status	Unmarried	11 (3.7)
	Married	265 (88.3)
	Divorced/widowed	24 (8.0)
Occupation	Working	216 (72.0)
	Not working	58 (19.3)
	Retired	26 (8.7)
Residence area	Urban	131 (43.7)
	Rural	169 (56.3)
Income (per month, RMB)	< 3,000	143 (47.7)
	3,000–5,000	126 (42.0)
	> 5,000	31 (10.3)
Insurance	New Cooperative Medical System	168 (56.0)
	Basic Medical Insurance for Urban Employees	83 (27.7)
	Basic Medical Insurance for Urban Residents	48 (16.0)
	None	1 (0.3)
Religion	No	282 (94.0)
	Yes	18 (6.0)
Cancer position	Head & Neck	51 (17.0)
	Intracranial	19 (6.3)
	Breast	62 (20.7)
	Respiratory system	71 (23.7)
	Digestion system	83 (27.7)
	Urinary/reproductive system	14 (4.6)
Cancer stage	Stage I	17 (5.7)
	Stage II	102 (34.0)
	Stage III	139 (46.3)
	Stage IV	42 (14.0)
Pain	No	174 (58.0)
	Yes	126 (42.0)

### Descriptive statistics and correlation analysis

Table 2 shows the descriptions and correlations of major variables. The mean ± standard deviation scores for EA, MLQ-P, MLQ-S, and DA were 24.91 ± 9.654 points, 24.91 ± 4.895 points, 19.19 ± 4.236 points, and 7.74 ± 4.176 points, respectively. According to the rule of thumb for interpreting the size of a correlation coefficient, a coefficient from 0.1 to 0.3 was weak correlations, and 0.4 to 0.6 was moderate correlations [36]. MLQ-P (*r* = 0.520, *p* < 0.01) was moderately positively correlated with MLQ-S. MLQ-P was moderately negatively correlated with both DA (*r* = -0.400, *p* < 0.01) and EA (*r* = -0.408, *p* < 0.01). While MLQ-S was weakly negatively correlated with DA (*r* = -0.151, *p* < 0.01) and EA (*r* = -0.142, *p* < 0.05). EA was moderately positively correlated with DA (*r* = 0.552, *p* < 0.01).

**Table 2:** Descriptive statistics and correlation analysis

Variable	1	2	3	4	Mean	SD
1. EA	1				24.91	9.654
2. MLQ-P	−.408^a^	1			24.91	4.895
3. MLQ-S	−.142^b^	.520^a^	1		19.19	4.236
4. DA	.552^a^	−.400^a^	−.151^a^	1	7.74	4.176

*Abbreviation*: SD, standard deviation

^a^*P <* 0.01, ^b^*P* < 0.05

**Table 3:** Hierarchical regression analysis

Model	Dependent	Predictors	Model summary			Coefficients			
			*F*	*R* ^*2*^	*B*		*SE*	*β*	
1	DA	EA	130.51^a^	0.31	0.24		0.02	0.55^a^	
2	MLQ-S	EA	6.171^b^	0.02	−0.06		0.03	−0.14^b^	
3	MLQ-P	EA	92.80^a^	0.39	−0.17		0.02	−0.34^a^	
		MLQ-S			0.55		0.05	0.47^a^	
4	DA	EA	51.25^a^	0.34	0.20		0.02	0.46^a^	
		MLQ-S			0.03		0.06	0.03	
		MLQ-P			−0.19		0.05	−0.23^a^	

*Abbreviation*: SE, standard error

^a^*P*< 0.01, ^b^*P*< 0.05

### Hierarchical regression analysis

According to the procedures for mediating-effect analysis, a series of simple regression analyses (Table 3) indicated that EA (β = 0.55, *p* < 0.01) is a predictor of DA (model 1), EA (β = −0.14, *p* < 0.05) is a predictor of MLQ-S (model 2), and EA (β = −0.34, *p* < 0.01) and MLQ-S (β = 0.47, *p* < 0.01) are predictors of MLQ-P (model 3), respectively. Next, a multiple regression analysis was conducted to test the final step of hierarchical regression analysis, and the results show that EA (β = 0.46, *p* < 0.01) and MLQ-P (β = −0.23, *p* < 0.01) are predictors of DA. However, the relationship between MLQ-S and DA was insignificant (model 4). As for Wen’s procedures, if at least one of effect (a or b) was not significant, then the bootstrap test was conducted to test the indirect effect in the mediating model.

### Mediating effect analysis

**Fig. 1:**
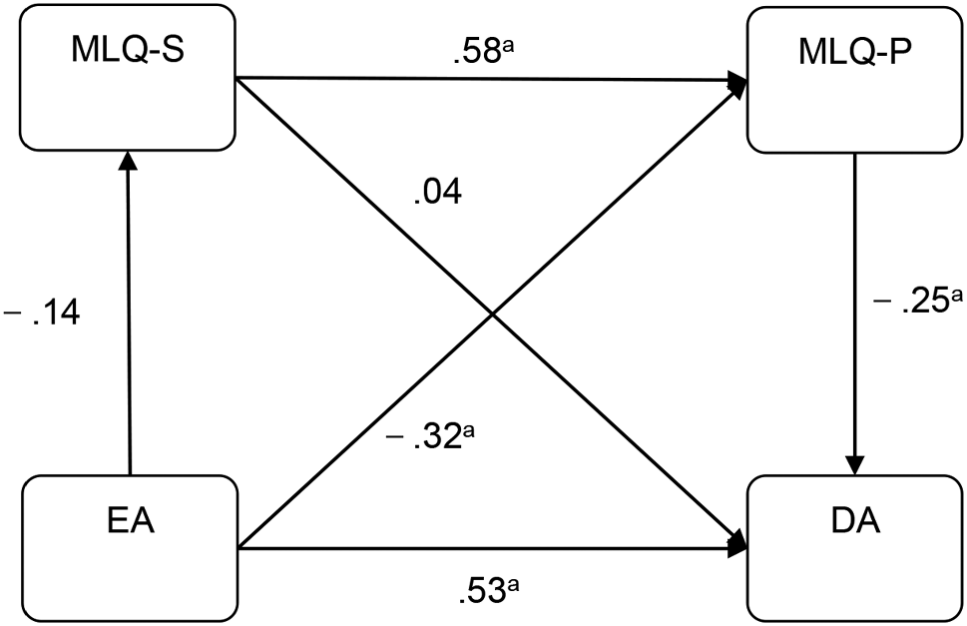
Crude model. *Note*: All the coefficients are standardized, ^*a*^*P < 0.01*, ^*b*^*P < 0.05*

**Fig. 2:**
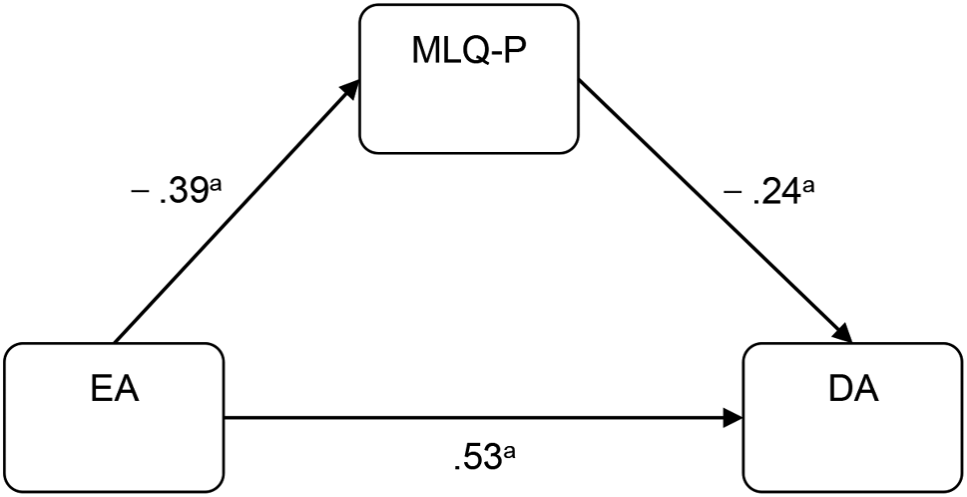
Final model. *Note*: All the coefficients are standardized, ^*a*^*P < 0.01*, ^*b*^*P < 0.05*

The mediation model was developed in IBM SPSS Amos version 23.0 and tested for significance using a bootstrap estimation procedure (2,000 bootstrap samples were specified). A partially mediated model containing indirect and direct paths from EA to DA was tested. The model fit indices of the crude model were poor (χ2 / df = 2.908, RMSEA = 0.080, GFI = 0.861, and NFI = 0.888, IFI = 0.923, TLI = 0.911, CFI = 0.923). In the crude model (Fig. 1), bootstrap results indicated that the direct paths were insignificant from EA to MLQ-S (95% confidence interval [CI], − 0.295 to 0.005) and from MLQ-S to DA (95% CI, − 0.212 to 0.005), respectively. Therefore, the mediating variable of MLQ-S was removed. The final model is presented in Fig. 2. This model had good fitting indices (χ2 / df = 2.524, RMSEA = 0.071, GFI = 0.908, and NFI = 0.928, IFI = 0.955, TLI = 0.944, CFI = 0.955), revealing that MLQ-P partially mediated the effect of EA on DA. Table 4 shows the direct and indirect effects of EA on DA and their associated 95% confidence intervals. The results indicate that all paths in the final model are significant. The mediating effect of MLQ-P accounts for 14.52% of the total effect.

**Table 4:** Direct and indirect effects of experiential avoidance on death anxiety

Model pathways	Estimated effect	Bias-corrected 95% CI		Percentile 95% CI	
		Lower	Upper	Lower	Upper
**Total effect**					
EA → DA	0.62	0.53	0.71	0.53	0.71
**Direct effect**					
EA →MLQ-P	−0.39	−0.51	−0.26	−0.51	−0.26
MLQ-P → DA	−0.24	−0.34	−0.14	−0.34	−0.13
EA → DA	0.53	0.42	0.63	0.42	0.63
**Indirect effect**					
EA →MLQ-P → DA	0.09	0.05	0.16	0.05	0.15

*Abbreviation* CI, confidence interval

## Discussion

This study explored the relationship between EA, MIL, and DA based on TMT and MMT theory in order to lay the foundation for the effective management of DA in cancer patients. To our knowledge, this is the first cross-sectional study to verify the predictor role of experiential avoidance to death anxiety in Chinese cancer patients, as well as the mediating effect of meaning in life among them. What follows is a discussion of the study findings.

### DA of Chinese cancer patients

The mean score of DA in this study was 7.74 points, consistent with that of other Asian cancer patients (M = 7.57; 95% CI, 6.58 to 8.56 from a meta-analysis) but relatively higher than those of populations in Europe (M = 6.47; 95% CI, 5.73 to 7.21) and North America (M = 5.57; 95% CI, 4.18–6.95) [10]. This finding suggests that Chinese cancer patients may experience a higher level of DA compared to patients of other ethnicities; however, a meaningful explanation for this difference appears to be lacking at this time. Possible influencing factors include religion, culture, income, insurance, and level of medical care [37, 38]. In addition, the factors of patients’ psychological attitudes and reactions to cancer stimuli are worth considering.

### The relationship between EA and DA in Chinese cancer patients

This study found that EA was moderately positively correlated with DA of cancer patients; specifically, the higher the EA score, the higher the DA. This finding is consistent with those of many previous studies and theories. A series of studies have pointed out that EA is associated with a variety of psychological problems and is a risk and maintenance factor for anxiety [39, 40]. Aqajani et al. conducted a descriptive study of 108 people and showed that cognitive fusion and avoidance were significantly positively correlated with DA [41]. Emerging evidence also suggests that if people can flexibly shift their attention from fear of stimulus to controllable emotions, the predictability of EA to anxiety will be reduced [42].

TMT, which describes the conflict between survival aspirations and death threats, may provide a reasonable explanation for this result [43]. According to TMT, when people realize the inevitability of death, their primary motivation is avoidance, and a key behavior is banishing these thoughts from their consciousness [44]. In TMT, EA falls under the umbrella of proximal defenses and involves denying death threats and anxiety through a variety of strategies [45]. For example, people with high levels of EA are more likely to use certain strategies, such as denial, avoidance, behavior disengagement, and self-blame, to deal with stressful situations like DA [46]. This repressive approach typically predisposes people to passively seek external support rather than actively face death and may lead to severe phobias and despairing behaviors. In contrast, people with lower levels of EA tend to use more psychological-adaptation strategies, such as active restructuring and acceptance, to deal with DA [19]. Thus, increasing an individual’s positive acceptance of death can reduce or eliminate their anxiety and negative emotions associated with death or near-death.

### The mediating effect of MIL between EA and DA in Chinese cancer patients

The results of this study also reveal the mediating effect of MIL between EA and DA. EA can predict DA of cancer patients through the presence of the meaning dimension of MIL. Patients with lower levels of EA typically find greater MIL and thus experience a lower degree of DA. These findings have been confirmed in other studies. Arslan et al. pointed out that EA is negatively related to MIL in a cross-sectional study, suggesting that psychological flexibility has a significant positive predictive effect on MIL [25]. Ardelt et al. also found that MIL was significantly negatively correlated with DA, reporting that individuals who felt more deeply about MIL were more likely to accept death and experience less DA [47]. Therefore, MIL can serve as a buffer between potentially traumatic events and negative outcomes and is the key to protecting and promoting mental health.

In TMT, avoidance and denial are proximal defenses, while MIL is considered a distal defense. The creation and maintenance of meaning is regarded as an instinctive defensive response to death threats, and its purpose is to minimize anxiety and fear [45]. According to MMT, death acceptance and MIL are the primary motivations for coping with DA. This theory provides the effective protection for overcoming the fear of death by actively accepting death and consciously transforming negative emotions about death into positive thoughts. More importantly, MMT also focuses on the tendency of human beings to live a meaningful life—that is, to find MIL [48]. These theories show that offense is the best defense. While recognizing the value of defense mechanisms, the most effective way to protect oneself from DA is to focus on living a dynamic and meaningful life. In other words, cancer patients can reduce their DA by reducing EA, enhancing death acceptance, and realizing and experiencing MIL.

### Limitations

Although this study provides important implications for research and practice, there are some limitations in its methodology. First of all, this study used a cross-sectional design, which could not determine the dynamic changes between the study variables at different disease stages. Future research should use longitudinal methods to test the proposed mediation model. Second, the study data were collected using self-reporting methods, which are insufficient means to reveal the physiological mechanisms of DA. Objective indicators, such as cortisol, could be considered in future studies [49]. Finally, participants in the study included 300 cancer patients recruited from a tertiary cancer hospital in Changsha, Hunan, China, and the limited representativeness of this sample may inhibit the generalization of the study results to other regions of China or the world. Future multicenter studies with further validation in different populations and settings are needed to explore the association between variables.

### Implications for practice

The results of this study provide a new path for studying the mechanism of DA and suggest a more positive and promising strategy for its management. Consistent with the concept of positive psychology, a gradual reduction in DA may benefit from a decrease in death avoidance, an increase in positive acceptance of death, and a proactive approach to creating and sustaining MIL. Khalvati et al. pointed out that behavioral and mental interventions based on death acceptance and MIL could be introduced to reduce patients’ DA [50]. Therefore, health care providers can adopt psychological interventions that promote acceptance and increase meaning, thus complementing and replacing interventions that promote defensiveness and transference. By consciously transforming negative thoughts about death into positive thoughts and by accepting and understanding death, cancer patients are motivated to build and find meaning in their lives in order to release their inherent potential and thus reduce DA.

## Conclusions

Our findings suggest that EA was a predictor of DA and that MIL was a significant mediator between EA and DA in Chinese cancer patients. Psychological interventions that promote acceptance and increase meaning should be developed that focus on helping cancer patients to overcome EA and enhance their acceptance may enable them to improve their MIL and further reduce DA.

## Data Availability

The data that support the findings of this study are available from the corresponding author upon reasonable request.

## Abbreviations

DA: Death anxiety
MIL: Meaning in life
EA: Experiential avoidance
TMT: Terror management theory
MMT: Meaning management theory
AAQ-II: Acceptance and Action Questionnaire II
MLQ: Meaning in Life Questionnaire
T-DAS: Death Anxiety Scale
χ2/ df: Relative chi-square
RMSEA: Root mean square error of approximation
GFI: Goodness-of-fit index
NFI: Normed fit index
IFI: Incremental fit index
TLI: Tucker–Lewis index
CFI: Comparative fit index
